# Health Behaviors Are Moralized When Perceived to Cause Harm

**DOI:** 10.1177/01461672251372823

**Published:** 2025-11-03

**Authors:** Samuel Pratt, Daniel L. Rosenfeld, Amelia Goranson, A. Janet Tomiyama, Paschal Sheeran, Kurt Gray

**Affiliations:** 1 University of California, Los Angeles, USA; 2 Knox College, Galesburg, IL, USA; 3 The University of North Carolina at Chapel Hill, USA; 4 The Ohio State University, Columbus, USA

**Keywords:** morality, health, attitudes, values, dyadic morality

## Abstract

People readily moralize health, whether by criticizing smokers or treating exercise as noble. Drawing from the theory of dyadic morality, we theorized that people moralize health most strongly when they perceive poor health as a source of suffering. Through five studies (total *N* = 2,055), we show that perceived harm can drive the moralization of health. We identified three types of harm—personal, interpersonal, and collective—that people perceive as relevant to health and created a 15-item measure to capture each (Study 1). Perceived interpersonal harm reliably predicted moralizations of health, whether health was conceived broadly (Study 2) or as a concrete health issue (e.g., smoking, eating healthfully, disease prevention; Study 3). Experimentally manipulating the interpersonal harmfulness of a health behavior caused participants to moralize it (Studies 4 and 5), whereas disgust had no unique effect (Study 4). We suggest that perceived harm plays a key role in moralizing health.

People often debate the morality of health behaviors, including getting vaccinated (Lyons, 2014), using condoms (Sarkar, 2008), and reforming nutrition standards (Harris, 2015). Understanding how health behaviors become moralized—imbued with “moral relevance” (Rhee et al., 2019, p. 3)—can shed light on attitude polarization (e.g., about vegetarianism; Feinberg et al., 2019), explain social stigma (e.g., against obesity; Ringel & Ditto, 2019), and suggest interventions for promoting public health and positive behavior change. We present five studies suggesting that health behaviors become moralized when they are perceived as causing harm.

## What It Means to Moralize Health

Moralization refers to the process by which preferences that previously lacked moral value become converted into values—and therefore imbued with morality (Rozin, 1999; Rozin et al., 1997). The distinction between moral and nonmoral attitudes is significant because moralized attitudes feel objectively true (Goodwin & Darley, 2008), give rise to outrage (Hutcherson & Gross, 2011) and the desire to punish (Shultz et al., 1986), and are more resistant to change (Luttrell & Togans, 2021).

Drawing on these perspectives, we define the moralization of health as treating a health-related issue as worthy of moral concern. For example, a person who moralizes cigarette smoking should view the decision to smoke as a moral question of right or wrong, rather than a matter of personal preference. The scope of health moralizations is wide (Thomas, 2019), and so we define health broadly to include one’s general health status (e.g., being healthy versus unhealthy), structural factors that affect one’s health status (e.g., having access to health insurance), and specific behaviors that impact the health of an individual (e.g., eating healthfully) or others (e.g., wearing a mask). We draw from a harm-based theory of moral judgment (Schein & Gray, 2018) to suggest that diverse health issues each become moralized through perceptions of harm.

## The Role of Perceived Harm

History reveals many health behaviors that became moralized when viewed as harmful (Gusfield, 1984; Whorton, 2014). In the 1800s, for example, Dr. Adam Clarke claimed in his guide to human health that “neither the plague, nor war, nor small-pox, nor similar diseases, have produced results so disastrous to humanity as the pernicious habit of onanism” (i.e., masturbation; quoted in Kellogg, 1890, p. 233). On the basis of this perceived harm, Clarke saw masturbation as immoral.

In modern times, discourse about the morality of smoking, drinking, vaccination, sexual activity, and even body weight often focuses on perceived harm. For example, smoking cigarettes was long seen as a matter of personal choice and only became truly moralized—and morally condemned—when it became clear that secondhand smoke harms children (Rozin, 1999). Similarly, individuals who morally oppose obesity often argue that being overweight causes widespread suffering (Ringel & Ditto, 2019), whereas the “body positivity” movement argues that stigmatizing obesity is the true source of harm (Leboeuf, 2019)—in this case, competing perceptions of harm seem to moralize the issue in different ways. Relatedly, moral proponents of vaccination argue that it prevents harmful diseases (e.g., measles) whereas opponents argue that vaccination causes autism in children (Thomas, 2019).

Supporting these examples, over a decade of research suggests that moral judgments are typically rooted in perceptions of harm (Gray & Pratt, 2025). The Theory of Dyadic Morality (Schein & Gray, 2018) formalizes the link between harm and morality, proposing that moral judgments revolve around a psychological template involving a moral agent (i.e., a wrongdoer) causing intentional harm to a vulnerable victim. According to this perspective, the more an act seems to match this template, causing intentional harm to a victim, the more it is moralized. This explains why child abuse is moralized more strongly than double-parking—unlike bad parking, abuse involves clear harm to a vulnerable victim (Gray & Kubin, 2024). Of course, unhealthy behaviors are seldom performed with the intention to cause harm, and are accordingly moralized less than acts like child abuse, but the Theory of Dyadic Morality argues for a continuum of moral condemnation—and perceived moralization. The theory suggests that the more someone sees a health-related behavior as generally causing harm to a target, the more they should moralize that behavior.

## Challenges to Harm-Based Moralization

Ample research documents the central role of perceived harm in attitude moralization (D’Amore et al., 2022; Feinberg et al., 2019; Wisneski et al., 2020), but some research argues that harm plays a lesser role in moralization (Skitka et al., 2018). Related work argues that perceived harm is more a consequence than a cause of moral condemnation (Brandt et al., 2015; Haidt, 2001), with moralization being driven by perceptions of impurity and feelings of disgust (Haidt et al., 1993; Rozin, 1999). Though visceral feelings of disgust might feel connected to morality, one pharmacological study suggests they do not causally drive moral condemnation (see Gray et al., 2022). Supporting this, a meta-analysis finds that inducing incidental disgust (e.g., by exposing participants to a bad odor) does not increase moral condemnation (Landy & Goodwin, 2015). After all, many acts are disgusting but not immoral (e.g., cleaning up a diaper). Disgusting acts only become moralized when they seem intuitively harmful (e.g., donating HIV-contaminated blood; Schein et al., 2016).

Critiques of the idea of harm-based morality often define harm very narrowly—restricting it to objective physical violence or emotional damage (Haidt, 2012)—but ample psychological and anthropological evidence suggests that harm is a subjective, pluralistic perception that varies across people, cultures, and contexts (for a review see Gray & Pratt, 2025). Past work finds that even “harmless” wrongs (e.g., sexual depravity) can be seen as harmful in at least three ways—harming the self, specific others, or society at large (Gray et al., 2014). In one study, participants reported that defiling a dead animal (an ostensibly harmless “purity” violation) could harm the perpetrator (35% of participants), the animal (40%), or the broader society (5%) (Gray et al., 2014).

In this view, moral differences between individuals can reflect differences in who people perceive as being harmed, rather than reliance on different moral mechanisms. Similarly, health behaviors could become moralized when perceived as causing different kinds of harm: to oneself (e.g., smoking harms the future self), to specific others (e.g., secondhand smoke harms kids) or to society (e.g., relief for smoking-related diseases is burdensome to taxpayers). It is important to note that although “harm can mean many things . . . the paradigmatic immoral harm is interpersonal harm” (Gray & Pratt, 2025, p. 677). This seems to suggest that harm to specific others—rather than harm to the future self or society at large—should mostly strongly predict moralization.

Health behaviors and morality are complex and multifaceted, and there are likely a wide range of emotional processes (Smith et al., 2021) and motivational factors (Feinberg et al., 2019) that shape the moralization of health. However, we suggest that perceptions of harm are a relatively underexplored driver of the moralization of health. Past research has focused on the importance of disgust (e.g., Skitka et al., 2018) and explored whether behaviors can be moralized by being paired with incidental, unrelated harm (e.g., exposing participants to a picture of a cut finger; Wisneski & Skitka, 2017). Here we explore whether *intrinsic* perceptions of harm—perceptions that a behavior itself is harmful—predict the moralization of health.

## Overview of the Current Research

Five studies explored whether perceived harm predicts the moralization of health. Study 1 developed a scale to map perceptions of harm in the health domain. This study identified three types of harm (personal, interpersonal, and collective) that people perceive as consequences of poor health and provided a consistent measure for assessing perceived harm in subsequent studies. Studies 2 and 3 explored whether perceiving poor health (e.g., unhealthy lifestyles, poor health status) as harmful predicts the extent to which people moralize health in general (Study 2) and specific health issues (Study 3). Studies 4 and 5 examined whether experimentally manipulating the interpersonal harmfulness of a health behavior caused participants to moralize it more.

In each study, we report all manipulations, measures, and exclusions, as well as all preregistered procedures and analyses, when applicable. Materials, data, analysis scripts, and codebooks for all studies are available at https://osf.io/kzxgt/?6eb897154d5d440b910b24181bfc1385.

## Study 1: Different Types of Perceived Harm

Before exploring the link between perceived harm and the moralization of health, we developed a face-valid measure assessing perceptions of health-related harm. Drawing from past work (Gray et al., 2014), we conceptualized three potential victims: the self (i.e., personal harm), others (i.e., interpersonal harm), and society (i.e., collective harm). This approach mirrors other models of moral cognition, such as the Model of Moral Motives, which proposes that moral behaviors—whether harm-related or not—are often directed toward the self, others, or the broader group (Janoff-Bulman & Carnes, 2018). First, *personal* harm involves behaviors and conditions that increase one’s own likelihood of future suffering (e.g., smoking cigarettes)—and the future self is sometimes seen as a somewhat “different” person (Hershfield, 2019; Pronin et al., 2008). Second, *interpersonal* harm involves inflicting damage on specific others, especially vulnerable targets (e.g., secondhand smoke hurts kids). Being unhealthy itself may be moralized for its potential to cause interpersonal harm—for example, if someone becomes so unhealthy that they struggle to care for their loved ones. Third, *collective* harm centers on societal welfare. If people see health behaviors as harming society (e.g., obesity costing taxpayers millions), they may moralize the behavior.

First, we developed 21 initial items intended to assess perceptions of personal, interpersonal, and collective harms related to poor health. Next, we conducted a parallel analysis to identify the number of distinct factors present across these items, with each factor indicating a distinct way in which people perceive poor health as harmful. Finally, we conducted an exploratory factor analysis (EFA) extracting this indicated number of factors, which enabled us to characterize and measure the types of harm people perceive in poor health.

### Method

This study’s sample size, materials, exclusion criteria, and analyses were preregistered at https://osf.io/qa926/?a2bb244561384ea6a8e8afcdb1b4cb56.

#### Participants

Based on MacCallum et al.’s (2001) recommendations for optimal EFA sample size, we recruited a total sample of 500 participants to ensure ample power. Participants were U.S. adults recruited via Prolific. After excluding two participants who failed an attention check in the survey, 498 participants (240 men, 237 women, 21 nonbinary, *M*_age_ = 34.73, *SD* = 14.43) were retained for analyses.

#### Materials and Procedure

##### Perceived Harm

Participants responded to a set of 21 items (see Table 1) on scales ranging from 1 (*strongly disagree*) to 7 (*strongly agree*). These candidate items were intended to assess perceptions of personal, interpersonal, and collective harms related to poor health, with seven items designed for each perception.

**Table 1.** Factor Loadings of Items Assessing Perceived Harms of Poor Health in Study 1.

Item	Mean (*SD*)	Factor 1: personal harm	Factor 2: interpersonal harm	Factor 3: collective harm
If I don’t take care of my health, then I will suffer in the future.	6.12 (0.94)	**.87**	-.16	-.01
If I am unhealthy, then I am more likely to experience pain throughout my life.	5.79 (1.09)	**.83**	-.11	.05
If I am unhealthy, then I experience a great deal of suffering.	5.42 (1.18)	**.82**	-.01	.00
The unhealthier I become, the worse my life will be.	5.57 (1.27)	**.79**	-.03	.04
Being unhealthy is fundamentally harmful to who I am as a person.	5.26 (1.40)	**.60**	.18	.06
Living an unhealthy lifestyle weakens who I am as a person.^*^	5.04 (1.54)	**.43**	.29	.14
Living an unhealthy lifestyle is the most harmful thing I could possibly do to myself.^*^	4.86 (1.61)	**.41**	.50	-.23
Living an unhealthy lifestyle is the most harmful thing I could possibly do to the people I care about.	4.22 (1.81)	-.07	**.91**	-.11
Unhealthy people tend to act harmfully toward other people in general.	3.44 (1.64)	-.21	**.79**	.10
Unhealthy people bring pain and suffering to the people around them.	4.25 (1.58)	-.09	**.74**	.16
Causing someone else to become unhealthy is the most harmful thing one could possibly do.	4.59 (1.69)	.01	**.69**	-.02
Unhealthy people set a harmful example for those around them.	4.81 (1.49)	.07	**.67**	.04
If I don’t take care of my health, then other people in my life suffer as a result.^*^	5.24 (1.42)	.10	**.47**	.08
If I am unhealthy, then I am unable to care for people in my life.^*^	5.59 (1.30)	.18	**.46**	-.01
Unhealthy living conditions weaken society.	5.68 (1.31)	.03	-.17	**.84**
When society is unhealthy, the economy suffers.	5.49 (1.38)	.08	-.10	**.76**
A country can only be as strong as its citizens are healthy.	5.29 (1.35)	-.08	.12	**.70**
Unhealthy people make society weaker.	4.72 (1.64)	-.11	.26	**.65**
An unhealthy workforce is an unproductive workforce.	5.57 (1.38)	.10	.02	**.60**
Living an unhealthy lifestyle is harmful to society.^*^	4.93 (1.48)	-.04	.31	**.59**
The more diseases that spread across society, the weaker the economy gets.^*^	5.56 (1.26)	.15	-.06	**.59**
Eigenvalue		3.62	3.99	3.38
Proportion of variance explained		.17	.19	.16

*Note*. Loadings for each item’s intended factor are displayed in bold. Poorly performing items are indicated by an asterisk and were dropped from the scale.

### Results and Discussion

A parallel analysis suggested that a 3-factor structure best characterized the 21 items, as we expected. Following our preregistration, we conducted an EFA, using maximum likelihood factoring and promax rotation, extracting three factors (see Table 1). Items loaded clearly onto factors capturing perceptions of personal, interpersonal, and collective harms.

Using our preregistered item retention criteria—which entailed retaining items with coefficients that load at values of .50 or more onto their primary factor with no coefficients on other factors within .20 of the primary loading—we dropped two items from the personal harm subscale, two items from the interpersonal harm subscale, and zero items from the collective harm subscale. Overall, we observed strong item performance across subscales, with very high item retention based on our *a priori* criteria. Accordingly, to provide a more parsimonious measure of perceived harm, we decided *post hoc* to increase our primary factor loading retention threshold from .50 to .60. This step led us to drop two items on the collective harm subscale with factor loadings between .50-.60. Consequently, our final scale of perceived harm was comprised of three 5-item subscales.

Internal consistencies were high for all three subscales, with α = .88 for personal harm, α = .86 for interpersonal harm, and α = .85 for collective harm. Correlations were *r* = .55 between personal and interpersonal harms, *r* = .56 between personal and collective harms, and *r* = .58 between interpersonal and collective harms, all *p* < .001. The mean value was above the neutral midpoint for all but one scale item (“Unhealthy people tend to act harmfully toward other people in general”), suggesting that people agree that poor health can cause harm to the self (*M* = 5.44), others (*M* = 4.59), and the group (*M* = 5.32).

This study provided a 3-factor scale for assessing the perceived personal, interpersonal, and collective harmfulness of poor health, with each subscale demonstrating strong factor loadings and high internal consistency. Each subscale also showed clear divergence from other subscales, as evidenced by the results of parallel analysis, a lack of cross-loading between retained scale items, and a high eigenvalue for each factor. Having established this scale as an informative measure of perceived harm in the health domain, Study 2 examined whether perceptions of harm to the self, others, and the group predict the moralization of health.

## Study 2: Moralizing Health in General

Study 2 used the three-factor health-harm measure developed in Study 1 to explore whether seeing harm in general unhealthiness predicted its moralization. We hypothesized that perceiving the effects of poor health as more harmful—to the self, others, or the group—would predict stronger moralization of health.

### Important Covariates

Some argue that feelings of disgust predict the moralization of health beyond perceptions of harm (Rozin, 1999; Wisneski & Skitka, 2017). We therefore included measure of disgust sensitivity as a covariate (Haidt et al., 1994). Other covariates included the Big Five factors of personality—openness, conscientiousness, extraversion, agreeableness, and neuroticism (Donnellan et al., 2006)—along with age, gender, race, ethnicity, education, and income.

### Method

This study’s sample size, materials, exclusion criteria, hypotheses, and analyses were preregistered at https://osf.io/sn7ur/?4054767549ea410d80296bd9033b320c.

#### Participants

Despite the strong effect of perceived harm on moral judgment (Schein et al., 2016), we decided to recruit 720 participants to obtain 80% power to detect small effects of *r* = .10 at α = .05, two-tailed. Participants were U.S. adults, recruited via Prolific. After excluding 20 participants who failed an attention check in the survey, 700 participants (339 men, 338 women, 23 nonbinary, *M*_age_ = 35.52, *SD* = 13.02) were retained for analyses.

#### Materials

##### Perceived Harm

Perceptions of personal harm (α = .89), interpersonal harm (α = .82), and collective harm (α = .85) related to poor health were assessed using the final 5-item subscales developed for these variables in Study 1. Responses ranged from 1 (*strongly disagree*) to 7 (*strongly agree*).

##### Moralization

Moralization of health was assessed by the following five items (α = .91), adapted from Feinberg and colleagues’ (2019) moralization scale: “To what extent do you feel that it is moral to be healthy?”; “To what extent do you feel that it is immoral to be unhealthy?”; “To what extent do you feel that health is a moral issue?”; “To what extent are your feelings about health deeply connected to your beliefs about ‘right’ and ‘wrong’?”; and “To what extent are your attitudes toward health a reflection of your core moral beliefs?” Responses ranged from 1 (*not at all*) to 5 (*extremely much*).

##### Covariates

*Disgust sensitivity* (α = .84) was assessed by the Disgust Scale—Revised (DS-R; Haidt et al., 1994, modified by Olatunji et al., 2007). Big Five factors of personality—*openness* (α = .80), *conscientiousness* (α = .74), *extraversion* (α = .84), *agreeableness* (α = .84), and *neuroticism* (α = .81)—were assessed by Donnellan et al.’s (2006) Mini-IPIP scales. *Age*, *gender*, *race*, *ethnicity*, *educational attainment*, and *income* were assessed in standard ways, with full details available in our online Materials file. Gender was dummy coded with man as the reference group and woman and other genders as comparison levels. Race was dummy coded with White as the reference group and Black, Asian, biracial/multiracial, and other race as comparison levels. Ethnicity was coded as Hispanic/Latinx (1) and not Hispanic/Latinx (0).

#### Procedure

After consenting, participants completed the measures of perceived harm and all covariates in a randomized order, followed by the measure of moralization.

### Results and Discussion

Through an ordinary least squares (OLS) regression, we regressed moralization on perceived personal harm, perceived interpersonal harm, perceived collective harm, disgust sensitivity, all Big Five personality traits, age, gender, race, ethnicity, education, and income.

As predicted, greater perceptions of interpersonal harm strongly predicted more intense moralization of health, *β* = .50, *p* < .001 (see Table 2), and accounted for 25% of the variance in moralization. Although we predicted that both perceived personal harm and collective harm would predict some moralization, they did not. Similarly, disgust sensitivity did not significantly predict moralization. Three covariates (conscientiousness, gender, and race) were significant predictors, but at very small effect sizes: no covariate explained more than 1% of the variance in moralization.

**Table 2.** OLS Regression Predicting Moralization of Health in Study 2.

Predictor	*b*	*SE b*	*β*	*p*
Perceived Personal Harm	0.00	0.04	.00	.987
Perceived Interpersonal Harm	**0.39** ***	0.03	.50	< .001
Perceived Collective Harm	0.05	0.04	.05	.225
Disgust Sensitivity	0.07	0.18	.01	.711
Openness	0.00	0.04	.00	.915
Conscientiousness	**0.12** **	0.04	.10	.003
Extraversion	-0.06	0.03	-.07	.059
Agreeableness	0.03	0.04	.02	.526
Neuroticism	-0.04	0.04	-.04	.250
Age	0.00	0.00	-.04	.281
Gender: Woman	**-0.14** *	0.07	-.07	.044
Gender: Other	-0.32	0.18	-.06	.070
Race: Black	-0.06	0.14	-.01	.661
Race: Asian	-0.02	0.10	-.01	.807
Race: Biracial/Multiracial	0.20	0.17	.04	.238
Race: Other	**0.45** *	0.22	.07	.038
Ethnicity	-0.03	0.12	-.01	.803
Education	0.01	0.03	.01	.730
Income	0.03	0.02	.04	.246

*Note*. Model R^2^ = .35. ***p < .001. **p < .01. *p < .05. Statistically significant values are indicated in bold.

These findings reveal that—as expected—perceived interpersonal harm predicted moralization, but—contrary to expectations—neither personal harm nor collective harm significantly predicted moralization. Still, these results align with classic (Killen & Smetana, 2015; Tisak & Turiel, 1988) and modern work (Decety & Cowell, 2018; Schein & Gray, 2015) highlighting the importance of interpersonal harm in moral judgment. These results also suggest that perceived harm may better predict moralization than disgust, but two obvious limitations to this study are worth noting. First, disgust was only measured via general disgust sensitivity, rather than something more intrinsic to health behaviors (a limitation we address in Study 4). Second, we only assessed moralization of health in general rather than the moralization of specific behaviors. Study 3 addresses this by examining a variety of concrete behaviors. Although we remained interested in the role of personal and collective harm, the findings of Study 2 prompted us to focus our predictions on the role of perceived interpersonal harm in moralizing specific health behaviors.

## Study 3: Moralizing Specific Health Behaviors

Study 3 tested whether perceiving poor health as personally, interpersonally, or collectively harmful predicts the moralization of real-world health issues. Based on the objectives of the U.S. Department of Health and Human Services’ Healthy People 2030 Initiative (Office of Disease Prevention and Health Promotion, 2020), we selected eight health issues that are of high social and cultural relevance in the U.S.: exercising regularly, eating healthfully, smoking cigarettes, smoking marijuana, gaining weight, getting a flu shot each year, staying home when feeling sick, and having access to health insurance. These issues varied in their apparent relevance to personal, interpersonal, and collective harms while still having the potential to evoke any type of perceived harm. For example, not getting a flu shot could make you sick in the future (personal harm), get others sick through contagion (interpersonal harm), or burden the health care system with sick patients (collective harm). We hypothesized that the association between perceived interpersonal harm and moralization of health observed in Study 2 would replicate across these specific health issues.

### Method

This study’s sample size, materials, exclusion criteria, hypotheses, and analyses were preregistered at https://osf.io/8xcve/?c848f21066924d2aabd5cd39daac804c.

#### Participants

In Study 2, we observed a large effect (*β* = .50) of interpersonal harm. In Study 3, we conservatively powered our sample to allow us to detect an effect one half of this magnitude (*β* = .25) with 80% power at α = .05, two-tailed. A power analysis indicated a minimum sample of 120 participants. We decided to recruit 200 U.S. adults via Prolific. After excluding four participants who failed an attention check in the survey, 196 participants (95 men, 98 women, 3 nonbinary, *M*_age_ = 37.51, *SD* = 14.85) were retained for analyses.

#### Materials

##### Perceived Harm

Perceptions of personal harm (α = .90), interpersonal harm (α = .86), and collective harm (α = .87) related to poor health were assessed as they were in Study 2.

##### Moralization of Health Issues

The moralization of the eight health issues was each measured separately with a 5-item scale similar to the scale used in Study 2. For example, for the scale assessing moralization of eating healthfully, the item in Study 2 that read, “To what extent do you feel that it is moral to be healthy?” was replaced with an item that read, “To what extent do you feel that it is moral to eat healthfully?” Full details presenting these scale items are available in our online Materials file. Responses to items in each scale ranged from 1 (*not at all*) to 5 (*extremely much*). Internal consistencies ranged from α = .93 to .95 across the eight scales.

#### Procedure

After consenting, participants completed the three subscales of perceived harm in a randomized order, followed by the eight measures of moralization in a randomized order.

### Results and Discussion

We conducted eight OLS regressions, one for each health behavior. In each regression, we regressed moralization on perceived personal harm, perceived interpersonal harm, and perceived collective harm. Seven of these eight tests provided support for our hypothesis (see Table 3). For every type of health-related moralization—except for that of health insurance access—perceived interpersonal harm positively predicted moralization, with effect sizes ranging from small-medium to large (smallest *β* = .21, largest *β* = .55). Moreover, across each of these seven health issues, neither perceived personal harm nor collective harm were significantly associated with moralization. Moralization of access to health insurance was an outlier to this trend, as perceived personal harm positively predicted this moralization while neither perceived interpersonal harm nor collective harm showed any association.

**Table 3.** Summary of Eight OLS Regressions Predicting Moralization of Health Issues in Study 3.

Outcome predictor	*b*	*SE b*	*β*	R^2^	*p*
Moralization of Exercising Regularly				.31	
Perceived Personal Harm	0.00	0.08	.00		.957
Perceived Interpersonal Harm	0.42***	0.06	.55		< .001
Perceived Collective Harm	0.02	0.07	.02		.806
Moralization of Eating Healthfully				.29	
Perceived Personal Harm	0.05	0.08	.04		.574
Perceived Interpersonal Harm	**0.39** ***	0.06	.50		< .001
Perceived Collective Harm	0.02	0.07	.02		.802
Moralization of Smoking Cigarettes				.24	
Perceived Personal Harm	-0.06	0.11	-.04		.575
Perceived Interpersonal Harm	**0.45** ***	0.07	.47		< .001
Perceived Collective Harm	0.09	0.09	.08		.305
Moralization of Smoking Marijuana				.08	
Perceived Personal Harm	-0.15	0.10	-.13		.131
Perceived Interpersonal Harm	**0.26** ***	0.06	.33		< .001
Perceived Collective Harm	-0.02	0.08	-.02		.843
Moralization of Gaining Weight				.29	
Perceived Personal Harm	-0.03	0.08	-.03		.714
Perceived Interpersonal Harm	**0.34** ***	0.05	.50		< .001
Perceived Collective Harm	0.08	0.06	.10		.181
Moralization of Getting a Flu Shot Each Year				.08	
Perceived Personal Harm	-0.06	0.11	-.05		.548
Perceived Interpersonal Harm	**0.18** *	0.07	.21		.012
Perceived Collective Harm	0.16	0.09	.16		.069
Moralization of Staying Home When Feeling Sick				.11	
Perceived Personal Harm	0.12	0.10	.10		.241
Perceived Interpersonal Harm	**0.17** *	0.07	.21		.011
Perceived Collective Harm	0.08	0.08	.09		.306
Moralization of Having Access to Health Insurance				.08	
Perceived Personal Harm	**0.43** ***	0.12	.32		< .001
Perceived Interpersonal Harm	-0.15	0.08	-.16		.052
Perceived Collective Harm	0.02	0.09	.02		.858

*Note*. ***p < .001. *p < .05.

Statistically significant values are indicated in bold.

Overall, these results replicate the findings of Study 2, which found that people moralize poor health (in general) when it seems to cause interpersonal harm. Here we found that people moralize specific health behaviors when they perceive poor health as a source of interpersonal harm. These behaviors include preventive health behaviors (exercise, healthy eating, getting a flu shot), risky health behaviors (smoking cigarettes), tabooed drug use (smoking marijuana), body appearance (weight gain), and active disease spread (staying home when sick).

Despite the consistency of results from the last two studies, questions remain about the *causal* impact of perceived harm on the moralization of health, prompting us to run two additional experiments. Given that personal and collective harm did not consistently predict moralization in studies 2 and 3, these experiments focused on the role of interpersonal harm.

## Study 4: The Moralization of Social Distancing

This study tested whether perceptions of harm and/or disgust led people to moralize a recently-salient real-world health behavior: attending public gatherings while sick. People frequently disagree about the extent to which socially distancing during illness is a personal preference or a moral imperative (Francis & McNabb, 2022). We suggest that differences in these attitudes depend on the extent to which the behavior is perceived as harmful.

This 2 (Harm: high, low) × 2 (Disgust: high, low) vignette experiment described someone attending a crowded community festival while sick. We manipulated the harmfulness (i.e., contagiousness) and disgustingness (i.e., visible symptoms) of their illness. We hypothesized that framing the illness as interpersonally harmful would cause individuals to moralize the behavior more than a control condition, and that perceived harm would mediate the effect of condition more strongly than disgust.

### Method

All data, code, and materials, as well as the preregistration for this study’s sample size, materials, exclusion criteria, hypotheses, and analyses are publicly available on the Open Science Framework: https://osf.io/kzxgt/?6eb897154d5d440b910b24181bfc1385.

#### Participants

A power analysis indicated a minimum sample size of 128 participants to detect a medium effect (f = 0.25) in a 2 × 2 between-subjects ANOVA with 80% power at α = .05. To account for potential attrition, we recruited 200 U.S. adults via CloudResearch Connect. After excluding four participants who failed an attention check in the survey, 196 participants (108 men, 83 women, 4 nonbinary, *M*_age_ = 40.09, *SD* = 11.63) were retained for analyses.

#### Materials

##### Vignettes

The vignettes used in the study are displayed in Table 4. The low harm, low disgust vignette served as a control condition.

**Table 4.** Vignettes Used in Study 4.

	Low harm	High harm
Low Disgust	Jordan decided to attend a large community festival hosted in their town. The festival was bustling with activity, with families gathered around food trucks, children laughing on carnival rides, and groups of friends chatting near the live music stage. Jordan had recently been diagnosed with an illness. A doctor had informed Jordan that they were not contagious and posed no risk of infection to others. Jordan had no visible symptoms and appeared completely normal. Jordan enjoyed wandering through the crowds, chatting with others, and taking in the vibrant atmosphere.	Jordan decided to attend a large community festival hosted in their town. The festival was bustling with activity, with families gathered around food trucks, children laughing on carnival rides, and groups of friends chatting near the live music stage. Jordan had recently been diagnosed with an illness. A doctor had informed Jordan that their illness was extremely contagious and could spread easily through close contact and airborne droplets. However, Jordan had no visible symptoms and appeared completely normal. Jordan enjoyed wandering through the crowds, chatting with others, and taking in the vibrant atmosphere.
High Disgust	Jordan decided to attend a large community festival hosted in their town. The festival was bustling with activity, with families gathered around food trucks, children laughing on carnival rides, and groups of friends chatting near the live music stage. Jordan had recently been diagnosed with an illness. A doctor had informed Jordan that they were not contagious and posed no risk of infection to others. However, Jordan’s body was covered with boils and rashes, some of which appeared swollen and oozing. Jordan enjoyed wandering through the crowds, chatting with others, and taking in the vibrant atmosphere.	Jordan decided to attend a large community festival hosted in their town. The festival was bustling with activity, with families gathered around food trucks, children laughing on carnival rides, and groups of friends chatting near the live music stage. Jordan had recently been diagnosed with an illness. A doctor had informed Jordan that their illness was extremely contagious and could spread easily through close contact and airborne droplets. Jordan’s body was also covered with boils and rashes, some of which appeared swollen and oozing. Jordan enjoyed wandering through the crowds, chatting with others, and taking in the vibrant atmosphere.

##### Perceived Harm

Participants rated two items on a 5-point Likert-type scale (1 = *Not at all*; 5 = *Extremely*): “This behavior is harmful to others”; “This behavior causes others to suffer.”

##### Perceived Disgust

Participants rated two items on a 5-point Likert-type scale (1 = *Not at all*; 5 = *Extremely*): “This behavior is disgusting”; “This behavior is gross.”

##### Moralization

We assessed moralization (α = .92) in a manner consistent with our previous studies. Participants rated three items on a 5-point Likert-type scale (1 = *Not at all*; 5 = *Extremely*): “To what extent do you feel that this behavior is a moral issue (An issue where your attitudes are based on moral values)?”; “To what extent are your feelings about this behavior deeply connected to your beliefs about ‘right’ and ‘wrong’?”; “To what extent are your attitudes toward this behavior a reflection of your core moral beliefs?”

#### Procedure

After consenting, participants were randomly assigned to read one of four vignettes manipulating the harmfulness and disgustingness of the health behavior (attending a crowded community festival while sick). After reading the vignette, participants completed the measures of harm, disgust, and moralization in a randomized order.

#### Pilot Study

We conducted a pilot study (*N* = 183) to confirm that the harm and disgust manipulations elicited perceived harm and disgust (the preregistration, data, materials, and code are available in the online materials). As expected, the harm and harm + disgust conditions were perceived as significantly more harmful than the control condition and the disgust condition, *p*s < .001, but were not significantly different from one another in perceived harmfulness, *p* = .57. Similarly, the disgust and harm + disgust conditions were perceived as significantly more disgusting than the control condition and the harm condition (*p*s < .001) but were not significantly different from one another in perceived disgust (*p* = .75).^1^ The harm condition was perceived as slightly more disgusting than the control condition (*p* < .001), suggesting that people perceive an asymptomatic but contagious disease as somewhat disgusting.

### Results and Discussion

**Figure 1.**
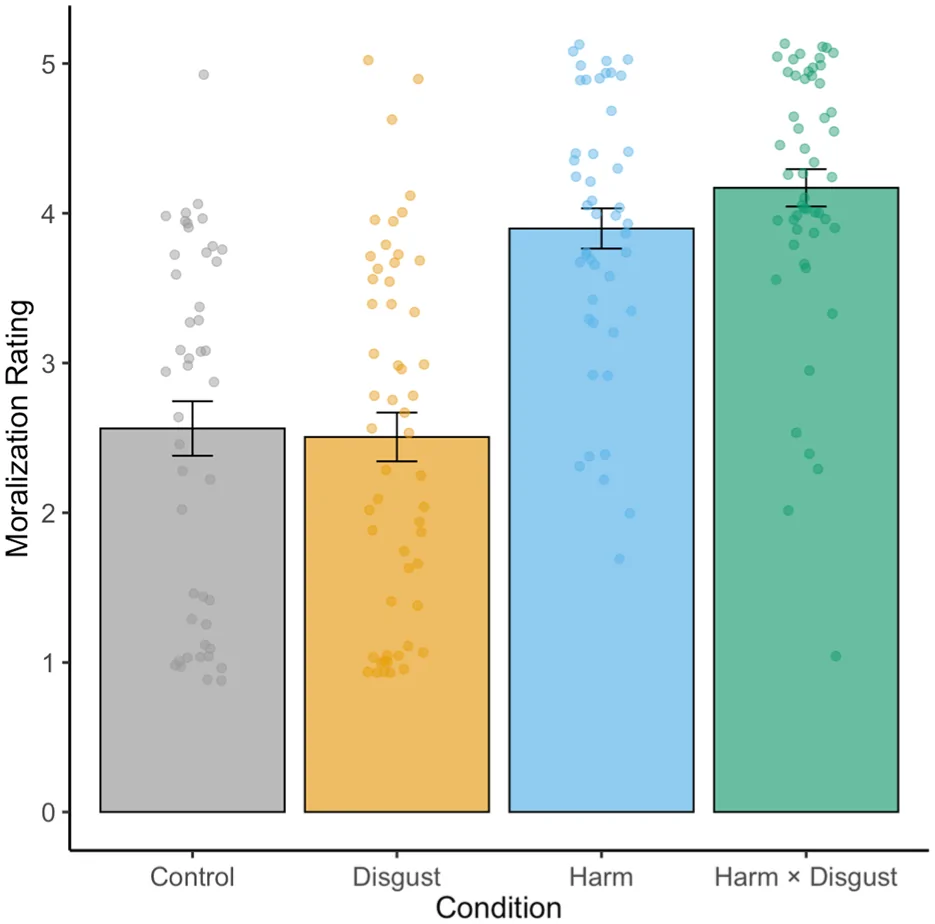
Mean Moralization Ratings by Condition in Study 4. *Note*. Error bars represent the standard error of the mean.

A 2 (Harm: high versus low) × 2 (Disgust: high versus low) factorial ANOVA revealed a significant main effect of harm, *F*(1, 192) = 98.01, *p* < .001, η² = .34, indicating greater moralization when the behavior was described as harmful. There was no main effect of disgust, *F*(1, 192) = 0.48, *p* = .489, η² = .002, and no significant harm × disgust interaction, *F*(1, 192) = 1.15, *p* = .284, η² = .004. In other words, framing the health behavior (attending a crowded festival while sick) as harmful, but not as disgusting, was sufficient to moralize it. Disgust only accompanied moralization when the behavior was also described as harmful (see Figure 1).

#### Mediation Analysis

**Figure 2.**
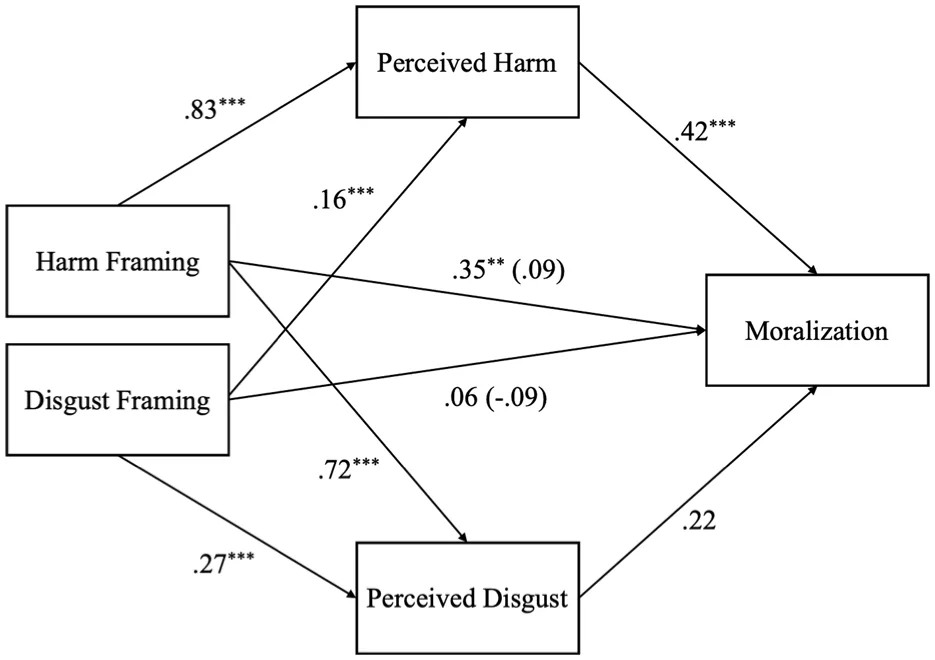
Perceived Harm Fully Mediates the Relationship Between Condition and Moralization (Study 4). *Note*. All path coefficients are standardized (β) ***p < .001. **p < .01.

Consistent with past research (Schein et al., 2016), we conducted a parallel mediation to examine the relative roles of perceived harm and perceived disgust in driving moralization (Figure 2). The lavaan package in R estimated a model where perceived harm and perceived disgust were simultaneous mediators. Experimental condition was represented by two contrast-coded predictors (harm condition and disgust condition). Standardized path coefficients were estimated using 5,000 bootstrap samples, which is standard practice for mediation (Preacher & Hayes, 2008).

The total indirect effect of condition on moralization via both mediators was significant, *b* = 0.52 (*β* = 0.41), *SE* = 0.11, 95% CI [0.29, 0.74], *p* < .001. However, when examining the mediators separately, we found that only perceived harm significantly mediated the effect of the manipulation. Specifically, the indirect effect through perceived harm was significant, *b* = 0.44 (*β* = 0.35), *SE =* 0.14, 95% CI [0.16, 0.73], *p =* .002, whereas the path through perceived disgust was not, *b* = 0.08 (*β* = 0.06), *SE* = 0.04, 95% CI [-0.01, 0.16], *p* = .085. The direct effects of both the harm condition (*b* = 0.11, β = 0.09, *p* = .262) and the disgust condition (*b* = -0.11, β = -0.09, *p* = .137) on moralization were nonsignificant after accounting for the mediators, suggesting full mediation, primarily through perceived harm. Notably, perceived harm alone accounted for about 86% of the total indirect effect of the experimental manipulation on moralization. Together, these results suggest that perceived harm, rather than disgust, was the primary driver of moralization.

These findings suggest that perceived interpersonal harm, rather than disgust, causally drives the moralization of health behaviors.^2^ There are many health behaviors that are disgusting (e.g., vomiting), but disgusting health behaviors only become *moral* issues when they are also interpersonally harmful (e.g., vomiting on a child; Schein et al., 2016). These results replicate those of our previous studies, but several limitations are worth noting. The first is that many people already moralize being in public while sick. Perhaps the purest test of moralization is whether a morally neutral behavior becomes moralized when it seems to cause harm (Feinberg et al., 2019; Rozin & Singh, 1999). The second limitation is that this study used a between-subjects design, but moralization is often discussed as a process that occurs over time within individuals (Rozin & Singh, 1999). To address these limitations, we conducted a final within-subjects experiment testing whether a morally neutral behavior becomes moralized when it is linked to harm.

## Study 5: The Moralization of Sleep Aids

Approximately 8% of Americans report regularly using sleep aid medication (Reuben et al., 2023). Could use of this medication—usually uncontroversial—become moralized when paired with perceived interpersonal harm? After measuring people’s baseline judgments of the morality of using sleep aids in a brief vignette, we manipulated whether the sleep aid use was described as *causing* harm (i.e., resulting in traffic deaths) or *preventing* harm (i.e., reducing traffic deaths) in a second vignette and measured people’s moral judgments again. Although prior research has focused primarily on the moralization of negative behaviors, less is known about the moralization of positive behaviors (e.g., viewing actions that prevent harm as morally virtuous). Although this type of positive moralization has been included in theoretical discussions (Rozin, 1999), little is known about the psychological mechanisms underlying positive moralization. Given the potential implications for promoting prosocial health behaviors, Study 5 offered a novel test of whether framing a behavior as harm-preventing can elicit moralization. We hypothesized that participants would moralize sleep aid use more when it was framed as causing or preventing harm. However, because moral violations are typically more salient than morally positive acts (Rozin, 1999), we expected the harm-causing condition to elicit stronger moralization than the harm-preventing condition.

To test the generalizability of these effects, we also varied whether the vignettes described someone starting sleep aids (i.e., an act of commission) or discontinuing sleep aids (i.e., an omission). Some health behaviors are moralized when actively performed (e.g., smoking) while others are moralized when omitted (e.g., not wearing a mask). We reasoned that if the perception of harm is a primary driver of moralization, it should moralize both actions and omissions.

### Method

All data, code, and materials, as well as the preregistration for this study’s sample size, materials, exclusion criteria, hypotheses, and analyses are publicly available on the Open Science Framework: https://osf.io/kzxgt/?6eb897154d5d440b910b24181bfc1385.

#### Participants

A power analysis indicated a minimum required sample size of 280 participants to detect a small effect (f = 0.10) in a 2 (Time: pre, post) × 2 (Action: adding, subtracting) × 2 (Consequence: harm-causing, harm-preventing) mixed ANOVA with 80% power at α = .05. To account for potential attrition, we recruited 300 U.S. adults via CloudResearch Connect. After excluding 18 participants who failed an attention check in the survey, 282 participants (151 men, 123 women, 8 nonbinary, *M*_age_ = 39.49, *SD* = 10.97) were retained for analyses.

#### Materials

##### Vignettes

Participants each read two vignettes. The first vignette described a person who begins (“commission”) or discontinues (“omission”) using sleep aids (Vignette 1). The second vignette described the consequences of the behavior as either causing harm (“harm-causing” framing) or preventing harm (“harm-preventing” framing; Vignette 2). The full wording of the vignettes is displayed in Table 5.

**Table 5.** Vignettes Used in Study 5.

Vignette 1	Vignette 2
***Commission (Starting Sleep Aids)*** Jordan had been dealing with sleep problems for some time and wanted to find a way to manage them. After doing some research, Jordan decided to start taking a sleep aid medication. Jordan followed the recommended dosage and took the sleep aid consistently each night. Over the next few weeks, Jordan continued with the new sleep routine.	Commission x Harm-Causing After taking the sleep aid, Jordan began feeling sluggish and unfocused in the mornings. Research shows that certain sleep aids can cause next-morning drowsiness, impairing driving ability in ways similar to being mildly intoxicated. **In fact, studies estimate that drowsy driving due to sleep aid use leads to 2,000 additional traffic deaths per year**. Jordan decides to continue using the sleep aid.
	***Commission x Harm-Preventing*** After taking the sleep aid, Jordan began feeling more rested in the mornings, with better focus throughout the day. Research shows that sleep aids can improve sleep quality, leading to better driving performance and reduced accident risk. **In fact, studies estimate that improved sleep quality from using sleep aids prevents 2,000 traffic deaths per year**. Jordan decides to continue using the sleep aid.
Omission (Stopping Sleep Aids) Jordan had been dealing with sleep problems for some time and wanted to find a way to manage them. After doing some research, Jordan decided to stop taking a sleep aid medication. Jordan followed the recommended process for discontinuing use and adjusted to sleeping without it over the next few weeks.	***Omission x Harm-Causing*** After stopping the sleep aid, Jordan began feeling sluggish and unfocused in the mornings. Research shows that stopping certain sleep aids can reduce sleep quality, impairing driving ability in ways similar to being mildly intoxicated. **In fact, studies estimate that reduced sleep quality from discontinuing sleep aids leads to 2,000 additional traffic deaths per year**. Jordan decides to continue to stay off the sleep aid.
	***Omission x Harm-Preventing*** After stopping the sleep aid, Jordan began feeling more rested in the mornings, with better focus throughout the day. Research shows that stopping certain sleep aids can eliminate next-morning grogginess, leading to better driving performance and reduced accident risk. **In fact, studies estimate that reduced drowsiness from discontinuing sleep aid use prevents 2,000 traffic deaths per year**. Jordan decides to continue to stay off the sleep aid.

##### Moralization

We assessed moralization (Time 1 α = .91; Time 2 α = .94) similarly to Studies 1-4. Participants rated three items on a 5-point Likert-type scale (1 = *Not at all*; 5 = *Extremely*): “To what extent do you feel that sleep aids are a moral issue (An issue where your attitudes are based on moral values)?”; “To what extent are your feelings about sleep aids deeply connected to your beliefs about ‘right’ and ‘wrong’?”; “To what extent are your attitudes toward sleep aids a reflection of your core moral beliefs?”

##### Moral Judgments

Participants rated the behavior on a single 7-point Likert-type scale with a neutral midpoint (1 = *Morally bad*; 7 = *Morally good*).

##### Manipulation Check

To confirm that the harm manipulation invoked perceptions of interpersonal harm, we gave participants the following question:

“Different behaviors can have a wide range of impacts on other people. Some behaviors harm people. Other behaviors help people. Other behaviors are neither harmful nor helpful to other people. Based on what you read, what impact does the behavior you read about have on other people?”

Participants responded on a 7-point Likert-type scale with a neutral midpoint (1 = *Extremely harmful*; 7 = *Extremely helpful*). An independent samples *t*-test confirmed that participants in the help condition viewed sleep aids as more helpful than participants in the harm condition, who viewed sleep aids as more harmful, *t*(554) = −18.48, *p* < .001, *M*_diff_ = −1.77, 95% CI [−1.97, −1.59].

#### Procedure

After consenting, participants were randomly assigned to read two sequential vignettes about a person who either starts or stops using sleep aids (Vignette 1) and whose behavior either causes harm or helps prevent harm to others (Vignette 2). Participants rated the morality of sleep aids and completed the moralization scale at two timepoints: immediately after reading Vignette 1 (Pre) and immediately after reading Vignette 2 (Post).

### Results and Discussion

**Figure 3.**
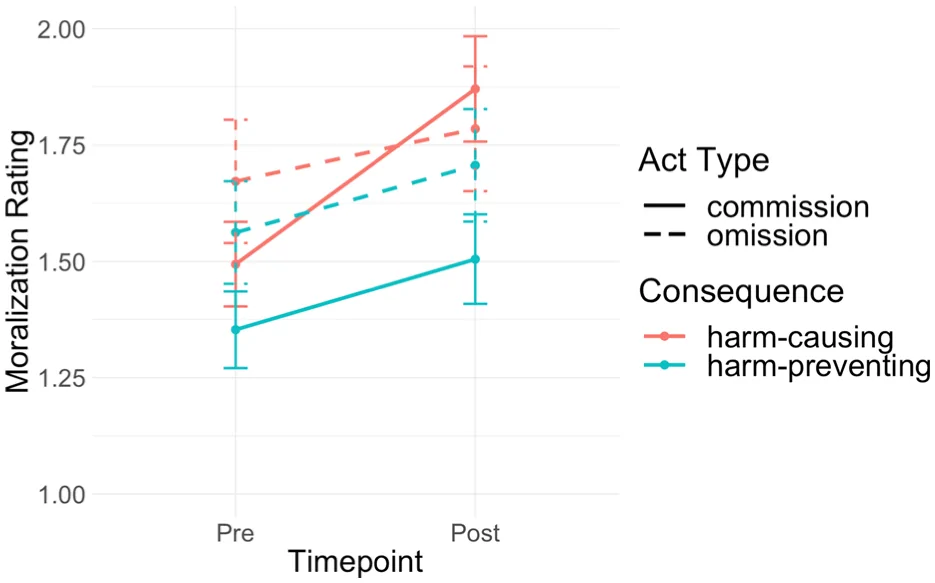
Framing a Health Behavior as Causing or Preventing Harm Causes Participants to Moralize it More. *Note*. Error bars represent the standard error of the mean. Scale of moralization rating is 1-5.

We conducted a 2 (Time: pre, post) × 2 (Action: commission, omision) × 2 (Consequence: harm-causing, harm-preventing) mixed ANOVA to examine whether moralization of sleep aids increased from premanipulation to postmanipulation when the consequences of the behavior were framed as harm-causing or harm-preventing, and whether this effect depended on whether the behavior was an act of commission (starting sleep aids) or omission (stopping sleep aids). There was a small but significant main effect of time, *F*(1, 278) = 28.23, *p* < .001, η²_G_ = .011, indicating that moralization significantly increased across all conditions from premanipulation to postmanipulation. There were no significant main effects of act type, *F*(1, 278) = 1.44, *p* = .23, η²_G_ = .005 or consequence, *F*(1, 278) = 2.76, *p* = .098, η²_G_ = .009, and no significant two-way or three-way interactions (all *p*s > .05), suggesting that the increases in moralization did not depend uniquely on whether sleep aids were framed as harm-causing or harm-preventing, nor whether they were framed as acts of commission or omission. To further examine changes within each condition, we conducted planned paired-samples *t-*tests comparing premanipulation and postmanipulation scores. Among all conditions, the largest increase in moralization from pre- to post- manipulation was observed when participants read about a harm-causing act of commission, *t*(83) = 5.22, *p* < .001, *M*_diff_ = 0.37, 95% CI [0.23, 0.52]. Overall, these results suggest that framing sleep aids as causing harm or preventing harm were both sufficient to moralize attitudes (see Figure 3).

**Figure 4.**
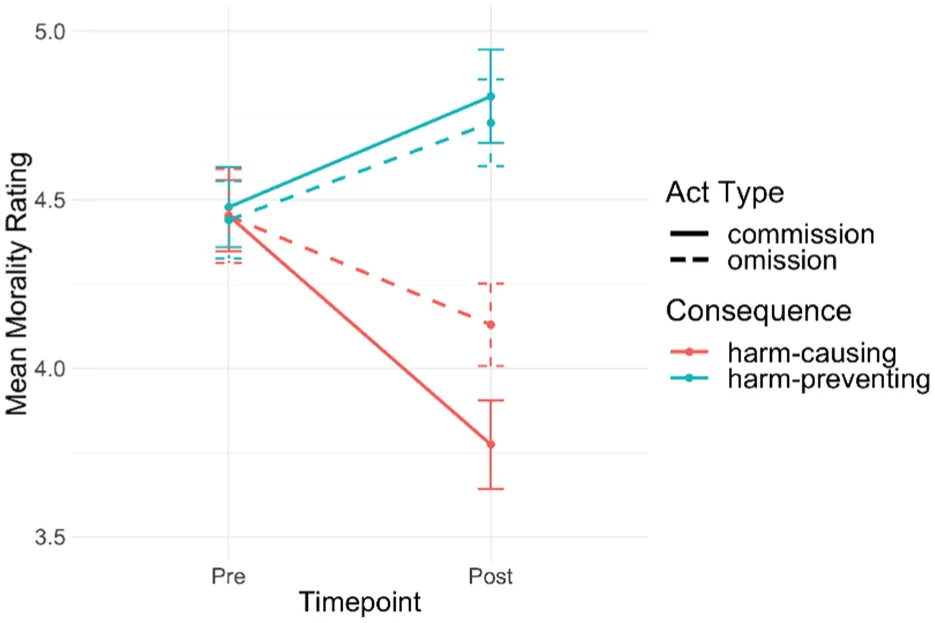
Framing a Health Behavior as Causing Harm Makes It Seem More Immoral. Framing a Health Behavior as Preventing Harm Makes It Seem More Moral. *Note.* Error bars represent the standard error of the mean. Morality was rated from 1 (morally bad) to 7 (morally good).

Secondary analyses on immorality judgments revealed a significant Consequence × Time interaction, *F*(1, 275) = 50.29, *p* < .001, η²_G_ = .036, suggesting that framing the consequences of sleep aids as either preventing harm or causing harm shifted the valence of moral judgments in the expected directions: framing the behavior as causing harm led participants to view it as more immoral, whereas framing the behavior as preventing harm made it seem more moral (see Figure 4). There was no significant three-way interaction, suggesting that the morality of causing or preventing harm did not depend on whether the act was one of commission or omission.

These results suggest that framing a morally neutral health behavior in terms of harm leads individuals to moralize it more. Participants did not initially moralize sleep aids (*M*_pre_ = 1.49 out of 5), but framing the consequences of sleep aids as either causing harm or preventing harm increased their moralization (*M*_post_ = 1.70). Though this effect was small, it is noteworthy that such a brief manipulation was sufficient to push participants’ attitudes toward greater moralization. In the real world, moralization likely builds through repeated exposure to salient messages (e.g., campaigns against marijuana), and this experiment demonstrates the moralizing effect of a single harm-related message. Importantly, framing sleep aids as preventing harm also increased moralization, suggesting that positive messaging can also be morally motivating. Perceived harm is a powerful moralizer of attitudes, whether it is used to facilitate positive health behaviors or prevent negative health behaviors.

## General Discussion

Through five studies, we investigated the link between perceived harm and the moralization of health. In Study 1 we developed a scale that mapped three types of harm related to poor health: personal (harm to the self), interpersonal (harm to others) and collective (harm to the group). Studies 2 and 3 found that the perception that poor health leads to interpersonal harm reliably predicts moralizations of health, both as a general concept (i.e., being healthy; Study 2) and specific health-related behaviors (e.g., smoking cigarettes; Study 3). Study 4 provided experimental evidence that framing a health behavior as harmful, but not as disgusting, causes it to become moralized. Study 5 found that framing a morally neutral behavior as harmful caused participants to moralize it more, and this effect generalized to actions, omissions, and a positive (i.e., harm-prevention) framing. Across studies, the more strongly participants perceived poor health or health behaviors as causing harm to others, the more inclined they were to view health issues as moral issues.

### Implications

Our findings advance a limited body of research on the moralization of health and help situate this work within existing models of moral cognition. People often moralize health behaviors, whether by viewing vaccination as sinful, stigmatizing obesity, or condemning cigarette smokers. These moralizations can lead to conflict and, in some cases, hinder public health (e.g., vaccine hesitancy). We suggest that understanding how people perceive harm in health behaviors can help make sense of individual differences in moralization.

The link between perceived harm and moralizations of health aligns with the Theory of Dyadic Morality, which argues that behaviors are judged as immoral to the extent that they seem harmful (Schein & Gray, 2018). The present research extends this theory within the health domain, suggesting that perceptions of interpersonal harm, but not personal or collective harm, are associated with moralizing health issues. Because interpersonal harms have more clearly identifiable victims compared to personal or collective harms, interpersonally harmful aspects of poor health may fit most directly within a dyadic template of moral judgment and thus be most influential for moralization.

We see the study of moral cognition as particularly valuable for understanding judgments about health and health issues. Moral beliefs are central to one’s sense of identity (Aquino & Reed, 2002; Strohminger & Nichols, 2014), and people tend to view moral beliefs as universal facts rather than subjective opinions (Goodwin & Darley, 2008; Skitka et al., 2005). At the individual level, this cognitive rigidity can create conflict and frustration when people with opposing moral viewpoints encounter others who think differently from themselves (Skitka et al., 2005). At the group level, moral norms and beliefs enable individuals to cooperate by establishing social order and prompting the prioritization of group interests above individual interests (Ellemers & Van Den Bos, 2012; Tomasello & Vaish, 2013). These characteristics of moral cognition yield both opportunities and hurdles for health promotion. When a health issue is moralized, the effects of conventional behavior change interventions may be attenuated because people are highly committed to their moral position and may be unlikely to weigh other factors (e.g., greater perceived benefits, self-efficacy, etc.) as much as they weigh their moral convictions. Moreover, when a health issue exhibits clear intergroup divides, such as that between liberals and conservatives during the COVID-19 pandemic, pressures to conform to moral norms of one’s in-group may inhibit behavior change.

At the same time, opportunities may exist in targeting perceptions of harm as a strategy to change individual health behaviors and attitudes. Moral attitudes are especially resistant to change (Luttrell & Togans, 2021), but research finds that individuals are more receptive to opposing moral viewpoints when those viewpoints are reframed in terms of one’s core values (Feinberg & Willer, 2019). Our findings provide an optimistic outlook in this regard: although people disagree widely about the morality of different health behaviors, a common value that unites disagreeing viewpoints is a concern about interpersonal harm and suffering. For example, debates about the morality of vaccine mandates often revolve around whether vaccines prevent harm or cause it (Geoghegan et al., 2020). To the extent that harm is a common concern across moral disagreements, reframing health issues in terms of the harm they cause or prevent to vulnerable individuals may be an effective way of bridging divides. For example, research finds that sharing one’s personal perceptions of harm builds respect across political disagreements (e.g., gun control debates), because both sides recognize the rationality of avoiding harm (Kubin et al., 2021).

Moralizations of health conditions and behaviors tend to evolve over time, as shown by shifting public and private opinions on topics like abortion (Saad, 2020) and smoking (Cummings & Proctor, 2014). As current health issues wax and wane in social/political prominence, attentiveness to perceptions of harm—especially interpersonal—could help to explain these evolutions. The results of our studies suggest that leaders may be able to influence public opinion by emphasizing or downplaying the extent to which health issues are interpersonally harmful.

### Future Directions

Our work helps integrate several theoretical perspectives in moral psychology, paving a path for future research. Moral psychology has long been divided about the precise roles of harm and gut feelings (e.g., disgust) in shaping moral judgments. Although we primarily approached this work through the lens of dyadic morality and harm, this theory does not deny the importance of these other influences, instead viewing them as integrated and mutually reinforcing (Gray et al., 2022). For example, prior research has identified disgust as an important factor in the condemnation of obesity (Ringel & Ditto, 2019). Other work finds that gut feelings of disgust serve as a cue to an act’s harmfulness, pushing people to see it as immoral (Gray et al., 2022). Although we did not find a link between disgust (e.g., disgust sensitivity, Study 3; perceptions of disgust, Study 4) and moralization in this work, there are opportunities for future researchers to further integrate these perspectives by examining the relationship between intuitive negative feelings and perceptions of harm in health-related issues.

Future work could also further explore moralization and the three types of harm investigated in Study 1—personal, interpersonal, and collective—which are similar to work on the Model of Moral Motives (Janoff-Bulman & Carnes, 2013). Although our studies did not find relationships between moralization and perceived harm at the personal or collective level, advocates of health behaviors and policies do often appeal to these kinds of harm (e.g., damage to one’s future self, or burdens on public health systems). On one hand, these appeals may simply be layered on top of concerns about interpersonal harm and so lack their own causal force. On the other hand, these appeals to personal or collective harm may lead to moralization when other conditions are met, such as invoking other normative values like self-restraint (for personal harm prevention) or social justice (for collective harm prevention).

Future research should also look more closely at individual differences in the moralization of health. Traits such as political ideology or the importance of morality to one’s identity may make some people more likely to moralize health than others (Feinberg et al., 2019; Graham et al., 2009). Our studies did not test whether these differences change how factors like perceived harm or disgust drive moralization. Understanding these differences could sharpen theories of moralization and help design better health interventions, such as those aimed at boosting vaccine uptake.

Importantly, moral cognition displays a negativity bias, where moral badness looms larger than moral goodness (Baumeister et al., 2001; Rozin & Royzman, 2001). This bias makes sense with health: evolution should prioritize the avoidance of sickness or death (Gluckman et al., 2011; Williams & Nesse, 1991). It therefore seems probable that *un*healthiness is a stronger impetus for moralization than healthiness. Interestingly, we found in Study 5 that emphasizing the positive (i.e., preventing harm) aspects of a health behavior was comparable to emphasizing negative aspects (i.e., causing harm) in terms of increasing moralization. Given the public health significance of promoting positive health behaviors, future research might consider shifting focus to positive moralizations, examining when and why perceptions of good health transform from morally neutral to morally virtuous. Moral praise for healthy eating, exercise, and infectious disease prevention are ripe for study, particularly in the context of social media and virtue signaling.

### Limitations

We employed a mix of observational and experimental methods, both within- and between-subjects, to investigate moralization from a variety of perspectives. However, one limitation of this work is that it does not assess the process of moralization over extended periods of time. Very few studies (e.g., Brandt et al., 2015; Feinberg et al., 2019) to date have investigated moralization as a longitudinal process, and more of such studies are needed. A second limitation is that because health is a large domain encompassing many specific conditions and behaviors, our studies can only examine some aspects of the moralization of health. Across studies, we sought to examine health widely in a variety of manifestations—including infectious disease, body weight, and as a general construct—but there is still much room to investigate moralization in the context of other aspects of health. A third limitation is that our research captured perceptions of harm and moralization as explicit attitudes, which could overlook potentially different patterns in implicit cognition. Finally, all participants in our studies resided in the U.S., which limits inferences about cross-cultural generalizability.

## Conclusion

From the doctor’s office to a politician’s desk, health issues are often imbued with morality. Our research suggests that people’s moralizations of health issues are largely driven by perceptions of interpersonal harm, such that the more strongly someone sees a health issue as causing harm to others, the more inclined they are to view that issue as morally significant. People see many health conditions and behaviors as having morally acceptable and punishable states, and disagreements over these matters can create intergroup divides, perpetuate social stigmas, and inhibit positive behavior change. Understanding why and how people perceive harm in health issues can help to explain disagreeing moral judgments and support efforts to promote public health.

## Notes

1. In the main study, the harm condition was rated as significantly more disgusting than the disgust condition and the control condition. Because participants were rating the disgustingness of the *behavior*, rather than the disgustingness of the *disease*, this pattern of data is consistent with the interpretation that participants were using “disgusting” and “gross” as moral terms synonymous with “wrong.” In the pilot study, when participants rated the disease alone, the harm condition was rated as less disgusting than the disgust condition, which is also consistent with this explanation.

2. Another possible explanation is that moralization in the high harm condition was driven in part by disobedience to authority. That is, the doctor telling Jordan that they were highly contagious might have been perceived as an implied directive to stay home; Jordan then attended a public gathering nonetheless. Though our data cannot speak to this possibility, we view this interpretation as consistent with harm-based moralization: disobeying the doctor’s orders would likely be perceived as immoral precisely because it could cause harm.
